# Two-Step *k*-means Clustering Based Information Entropy for Detecting Environmental Barriers Using Wearable Sensor

**DOI:** 10.3390/ijerph19020704

**Published:** 2022-01-08

**Authors:** Bogyeong Lee, Hyunsoo Kim

**Affiliations:** Department of Architectural Engineering, Dankook University, 152 Jukjeon-ro, Suji-gu, Yongin-si 16890, Korea; bglee_@dankook.ac.kr

**Keywords:** walkability, environmental barrier, *k*-means clustering, inertial measurement unit (imu), information entropy, wearable sensor

## Abstract

Walking is the most basic means of transportation. Therefore, continuous management of the walking environment is very important. In particular, the identification of environmental barriers that can impede walkability is the first step in improving the pedestrian experience. Current practices for identifying environmental barriers (e.g., expert investigation and survey) are time-consuming and require additional human resources. Hence, we have developed a method to identify environmental barriers based on information entropy considering that every individual behaves differently in the presence of external stimuli. The behavioral data of the gait process were recorded for 64 participants using a wearable sensor. Additionally, the data were classified into seven gait types using two-step *k*-means clustering. It was observed that the classified gaits create a probability distribution for each location to calculate information entropy. The values of calculated information entropy showed a high correlation in the presence or absence of environmental barriers. The results obtained facilitated the continuous monitoring of environmental barriers generated in a walking environment.

## 1. Introduction

Physical activity is important for both physical and mental well-being [1]. Among various physical activities, walking is the most basic activity [2,3]. It is also the most basic means of transportation. Additionally, walking allows people to stay healthy in their daily lives. An individual uses his body while walking resulting in direct interaction with the external environment [4]. In other words, a person’s walking is influenced by the external environment or built environment [5].

Considering that human behavior is affected by the conditions of the external environment while walking [6], the need to continuously manage the urban or built environment is increasing [7]. In particular, it is essential to provide a pedestrian-friendly walking environment. The term “pedestrian-friendly walking environment” may indicate an environment with high walkability [8]. The term “walkability” can be defined in various ways since the term includes many key themes or dimensions such as traversable environments, compact places, being safe for walking, physically-enticing environments, lively and sociable environments, an environment providing sustainable transportation options, and exercising-inducing environment [9]. Thus, walkability is determined by the combination of these dimensions. In addition, in the urban design domain, walkability cannot be measured by a just single dimension [10]. Although the concept “walkability” is very complicated because overall walkability of a specific street is the combination result of multi-dimensional features, it is clear that environmental barriers—an environmental feature makes pedestrians feel uncomfortable during walking—should be identified and eliminated to improve walkability [11,12]. There are diverse definitions of environmental barriers [13,14,15]. Even more, environmental barriers vary with age, gender, and socio-cultural contexts [15,16]. Environmental barriers in walking can include fixed (e.g., broken pavement, insufficient width, etc.) and unfixed features (e.g., vehicles, traffic volume, complexity, etc.) [10]. Considering that walking and gait are a result of an interaction between surfaces and human body, environmental barriers in this study are an environmental feature that can make pedestrians uncomfortable in a specific location (e.g., sidewalks and crosswalks). As shown in the previous study [17], this study uses a very narrow concept of walkability defined as how much regular gait patterns are maintained.

In existing research, many approaches (i.e., surveys by experts, surveys by actual users, reporting of complaints by civilians, walking audits, and walking interviews, etc.) have been developed to identify environmental barriers [18,19,20,21]. Although these methods have significantly improved the walking environment by identifying various environmental barriers, the following problems exist. Based on the results of surveys by experts, users of the actual walking environment are usually excluded from the evaluation stage. In addition, there is a possibility of subjective bias in the evaluation by experts [12], and continuous evaluation requires excessive financial expenditure [11]. However, in surveys by actual users, users of the walking environment can directly participate in the evaluation; however, a bias may exist depending on the sampling of people participating in the survey [22]. In addition, continuous monitoring is difficult because the evaluation of walking environment is performed at intervals [23]. Lastly, in the case of handling civilian complaints, the opinions of the complainants cannot be regarded as the opinions of the majority of pedestrians. Additionally, the opinions of civil petitioners can be prioritized in the budget execution process [24,25]. Summarizing the aforementioned problems: (1) Users of the walking environment are excluded from the evaluation process, (2) there is a possibility of subjective bias in the evaluation process, and (3) continuous evaluation is difficult due to problems such as budget and time.

Recent developments in sensing technology can provide an opportunity to solve the above-mentioned problems. People-centric sensing involves collecting and analyzing data with citizens as sensors [26]. In other words, it is possible to monitor various conditions (such as crowd detection, event detection, noise, traffic, etc.) of the built environment using data acquired from the daily lives of citizens [26,27,28,29,30]. People-centric sensing has also been used to identify environmental barriers. Existing studies have attempted to identify factors that cause discomfort by analyzing data generated from people’s daily lives [31,32,33].

The premise of existing studies is that there is a change in human behavior or response in the presence of external stimuli. Kim et al. [12] conducted a study to identify defects on sidewalks using pedestrian data collected from an inertial measurement unit (IMU). Zeile [34] developed a method to measure walkability by combining the data from surveys, biosensors, and geospatial analysis. Ahn et al. [35] conducted a study to identify environmental distress factors using electrodermal activity (EDA) and IMU. Kim et al. [36] proposed a method to evaluate the neighborhood built environment using EDA, gait patterns, and blood volume pulse. In addition, Lee et al. [23] conducted a study to determine environmental barriers based on the stress measured by a wearable sensor. Zanwar et al. [37] used wearable sensors to identify barriers that were not suitable for the elderly and disabled. Existing studies describe various methods to identify the factors that can inhibit walkability. However, these studies dealt only with the relationship between the point where the intensity of the pedestrian’s reaction is high and the existence of environmental barriers. In existing studies, the intensity of response means that the value of acceleration through the IMU is high [12], that the measured value of EDA appears high [35], and that the blood volume pulse is measured as high [36]. However, even at a point where the intensity of the reaction is low, an environmental barrier may exist. As shown in a previous study [38], when the floor is slippery in a walking environment, the value of acceleration through IMU can be measured as low by reducing the stride length or reducing the walking speed in response to this. Therefore, it is necessary to include these various reactions in the identification process of environmental barriers.

Human behavior is very complex, and the response to external stimuli (especially environmental barriers that can impair walkability) may differ for each individual. According to a study by Kim et al. [38], ironworkers showed various reactions while responding to hazards. The experimental results indicated that the strength of the reaction was negligible for a strong external stimulus. A notable point was that there were diverse gait patterns (low normality) regarding a construction hazard. On a slippery surface, some workers tried to slow down their gait speed, and others slipped. In another study for detecting defective sidewalk (finding cracks, holes, and vertical separations) [12], pedestrians also showed diverse responses to defects (low normality). On the other hand, pedestrians’ gait patterns were highly maintained under normal conditions (without sidewalk defects). In a study by Kim [39], when a car passes around a pedestrian on a road with a mixture of sidewalks and lanes, pedestrians show very low intensity of responses or stop and wait for the vehicle to pass. Conversely, there were pedestrians who took immediate action to avoid contact with vehicles. When environmental barriers act, the unpredictable characteristics of the collective responses of pedestrians is highly dispersed. According to information entropy theory, high unpredictability contains more information and has high entropy.

In many previous studies, entropy theory has been used to measure walkability [40]. Related studies calculated walkability by calculating entropy according to the degree of land use mix [41,42,43]. In addition, research in fields, such as crowd safety [44], emergence [45], pedestrian flow [46], evasive actions of pedestrians [47], and abnormal pedestrian detection [48] through the entropy of pedestrian behavior have also been conducted.

Considering the advantages of using wearable sensor and information entropy in existing studies and the irregular changes in the behavior of pedestrians at a specific location, the existence of an environmental barrier can also be estimated through the degree of entropy of the observed behavior. This study is conducted to achieve the following objectives: (1) developing an information entropy calculation method that can quantify pedestrians’ diverse responses; (2) testing the suggested method in an actual walking environment; (3) comparing the calculated information entropy values and environmental barriers; and (4) confirming that the method proposed in this study is feasible.

## 2. Methodology

### 2.1. Experimental Design

**Figure 1:**
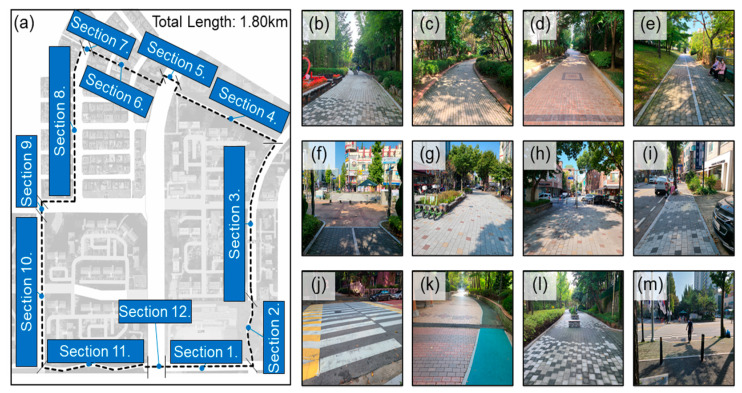
Overview of experimental site; (**a**) Site and sections and (**b**–**m**) representative pictures of each section.

To achieve the purpose of this study, a certain number of participants were recruited and wearable sensors were attached to them. They were instructed to walk in the experimental site. Figure 1 shows the experimental site used in this study. The total length of the test site is 1.80 km. It is divided into 12 sections according to the characteristics and continuity. The best representation of each section is shown in Figure 1b–m, and the order of each figure follows the section number.

The experimental site is a district in Bundang-gu, Seongnam-si, Gyeonggi-do, Korea. The reason for selecting this area is its diversity of walking environments. We have closely examined the actual pedestrian interaction in various environments and identified the environmental factors that cause various behavioral responses. The study area includes parks, well-maintained roads, crosswalks, residential areas, and commercial areas. In addition, it was expected that a sufficient number of participants could be recruited from the area to secure sufficient statistical significance because the experimental area is a residential area. Table 1 shows the description, length, and average width of each section.

A total of 64 participants were recruited to conduct the experiments. Participants of various age groups who were healthy enough to participate in the experiment were recruited from the sports clubs (running club and senior gateball club) around the experimental site. The age and gender of the participants are shown in Table 2. None of them had any discomfort while walking or any physical constraint in performing the walking experiment for a distance of 1.8 km.

Prior to the experiment, all participants were informed that the trial had been approved by the Institutional Review Board (IRB: DKU 2020-09-027) and that all data would be anonymized and used for research purposes only. The experiment was performed in two stages. In the first stage, the participants were instructed to wear an IMU sensor on their right ankle and walk at a comfortable velocity along a set route (starting at Section 1 and finishing at Section 12). In the second stage, the participant and experimenter walked along the path together to examine and record the points at which the participant experienced an environmental barrier during the first stage of the experiment. In addition, a sufficient break (10–20 min) was provided between the experiments to recover from fatigue. The recorded information was used to analyze the relationship between the existence of an environmental barrier and information entropy values. Because a trial (conducting a participant’s experiment) takes about one hour, the experiment was conducted for 16 groups of 4 individuals over 27 days from 4 October to 30 October 2021 (excluding weekends and rainy days). During the experiment, the temperature was 14–20 °C and humidity was 25–40%.

### 2.2. Research Framework

**Figure 2:**
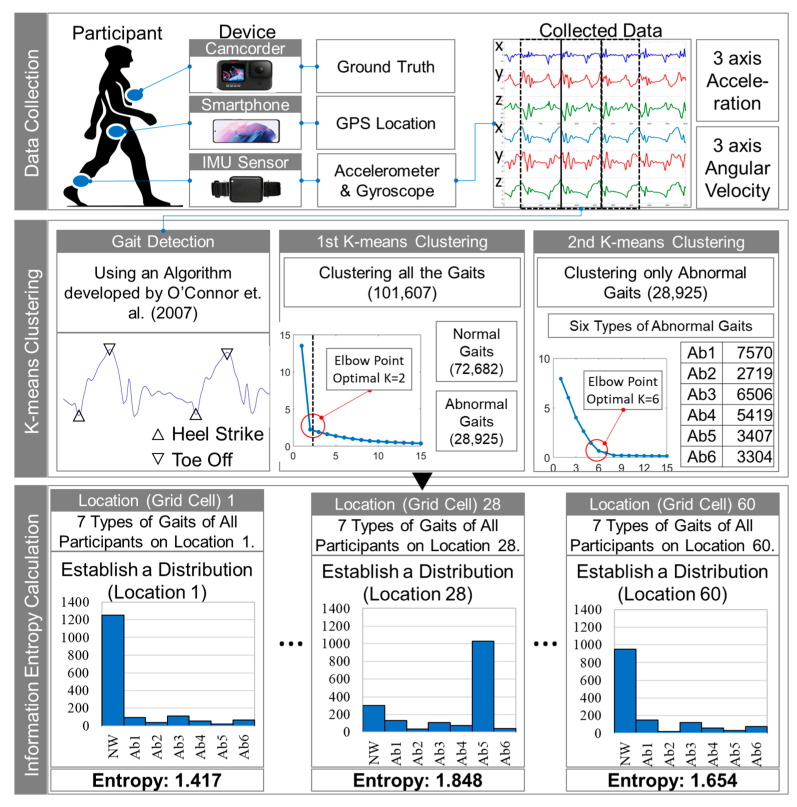
Research framework.

Figure 2 depicts the research framework proposed in this study. It consists of three major steps. First is data collection. Three devices are used to collect the data. First, the camcorder is used to confirm the ground truth and is attached to the participant’s chest. Second, a smartphone is placed in the front pocket of the participant’s trousers. Smartphones are used to collect GPS data during the experiment. Third, an IMU sensor is attached to the participant’s right ankle. During the experiment, the IMU sensor measures 3-axis acceleration and 3-axis angular velocity. In this study, data collected from camcorder and smartphone are not used to calculate information entropy but only for ground truth and GPS location.

The second step is to classify various gaits using *k*-means clustering. Before extracting individual gait features, gait detection should be performed on the 3-axis acceleration and 3-axis angular velocity data obtained through the IMU sensor. In this study, gait detection was performed using an algorithm proposed by O’Connor et al. [49]. Based on the six types of data collected from the IMU, the gait of each participant is detected based on heel strike and toe off. *k*-means clustering is used to distinguish between normal and abnormal gaits in the first clustering, and abnormal gaits are classified into six types in the second clustering.

Finally, the seven types of gait (one normal gait and six types of abnormal gait) are classified according to the measured location and a behavioral response distribution is created for each location. Based on this distribution, the information entropy for each section is constructed. The calculated information entropy values are verified against the location of the environmental barrier.

### 2.3. Calculation Process of Information Entropy by Location

As mentioned earlier, *k*-means clustering was performed twice in this study because two groups were classified (normal and abnormal gait) after the first clustering. To construct a detailed behavioral response distribution, we have re-clustered the groups classified as abnormal gaits in the first *k*-means clustering.

Since each detected gait includes various values, we have replaced six types of data with one value according to the gait. The average of the signal vector magnitude (*SVM*) is calculated to obtain the average of the intensities of six types of data appearing in one gait as represented in Equation (1):

(1) $$SVM_{ij}=\left[\overset{n}{\underset{k=1}{\sum }}\sqrt{x_{k}{}^{2}+y_{k}{}^{2}+z_{k}{}^{2}}\right]/n$$

where *n* is the total number of IMU measurements of the *j*th participant on the *i*th grid cell, *x_k_* is the *k*_th_ acceleration (or *k*th angular velocity) of the anterior-posterior axis, *y_k_* is the *k*th acceleration (or *k*th angular velocity) of the horizontal axis, and *z_k_* is the *k*th acceleration (or *k*th angular velocity) of the vertical axis.

The *SVM* values for each gait obtained using Equation (1) were normalized indicating that one gait has two normalized *SVM* values and clustering is performed based on these values. First *k*-means clustering is performed to classify gaits as normal and abnormal. Second *k*-means clustering is performed to classify only abnormal gaits. Finally, a behavioral response distribution is created based on seven types of gait.

The established behavioral response distribution by location is used to calculate information entropy. According to information theory, the entropy of a random variable is the average level of information [50]. This information is also the level of occurrence of an unexpected event [51]. Shannon [52] introduced the concept of information entropy to quantify the uncertainty of a random variable. In this study, the Shannon entropy (SE) can be represented by a combination of the probabilities of seven types of activity classified by two-step *k*-means clustering. SE can be calculated using Equation (2) as:

(2) $$H\left(A\right)=-\sum_{i=1}^{n}p\left(a_{i}\right)\log p(a_{i})$$

where *H*(*A*) is the entropy and *p*(*a_i_*) is the probability distribution.

## 3. Results

### 3.1. Environmental Barriers Identified by Participants’ Expression: Ground Truth

**Figure 3:**
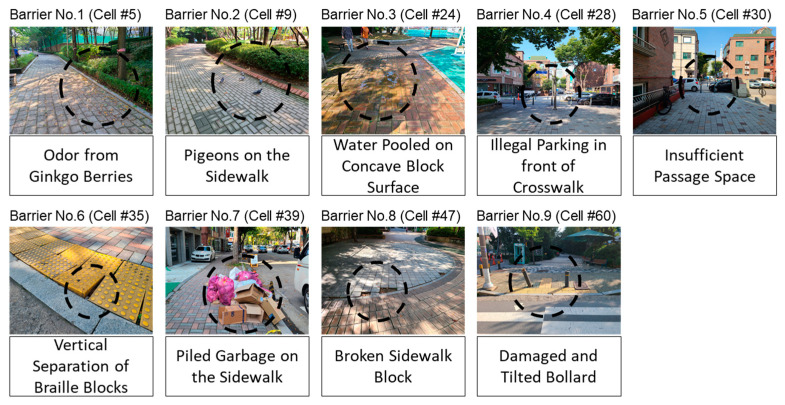
Captured environmental barriers as indicated by participants.

Figure 3 shows the specific points of environmental barrier as expressed by the participants in this study. After the first walking experiment, the participant and experimenter walked along the experimental route again and recorded the points indicated by the participant as an environmental barrier. To identify the environmental barriers indicated by 64 participants a point recorded as an environmental barrier more than six times (about 10% of the participants) was defined as an environmental barrier. Based on this, a total of nine environmental barriers were selected in this study.

The identified environmental barriers can be classified according to their occurrence characteristics and continuity as follows: (1) seasonal environmental barrier (barrier number 1), (2) temporal environmental barrier (barrier numbers 2 and 3), (3) human-made environmental barrier (barrier numbers 4, 5, and 7), and (4) physically disordered environmental barrier (barrier numbers 6, 8, and 9). Although the seasonal environmental barriers showed no significant difference from the surrounding environment, the participants tended to avoid it because of the odor of Ginkgo biloba. The temporal environmental barrier was not expressed as an environmental barrier by the participants if there was no pigeon or the water was not stagnant while performing the experiment. In other words, an element can act as an environmental barrier for a relatively short time. Human-made environmental barriers were due to illegal parking. However, the participants did not express discomfort when there was no illegal parking in the area. Lastly, the physical disordered environmental barrier was considered as an environmental barrier only by those who passed through the physical disorder. In the following sections, the process and feasibility of the identification of the above-mentioned environmental barriers using information entropy are explained.

### 3.2. Two-Step *k*-means Clustering Results

**Figure 4:**
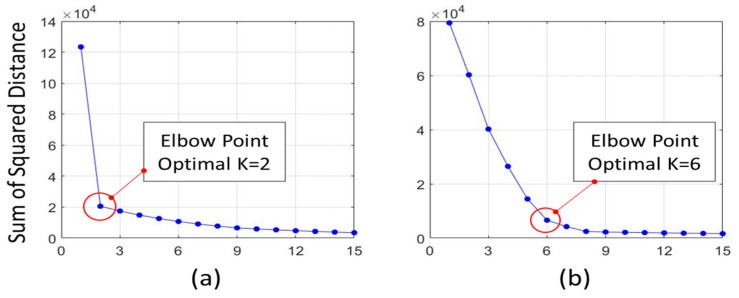
Optimal values of *k*-means clustering: (**a**) First clustering for all gaits and (**b**) second clustering for only abnormal gaits (excluding normal gaits).

In this study, the total number of gaits collected from 64 participants was 101,607. First, *k*-means clustering was performed for the entire gait. Next, the sum of the squared distances according to the value of *K* was calculated to obtain the optimal number of clusters. The optimal value of *K* was determined to be 2 based on the elbow point (see Figure 4a). When a *K* value of 2 was applied, the 72,682 gaits were classified into with normal gait, and 28,925 gaits were classified into abnormal gait.

Considering that people’s reactions to various external environments are also diverse, it is necessary to consider the reactions of pedestrians due to environmental barriers. Therefore, *k*-means clustering was performed again on only 28,925 abnormal gaits classified previously. The optimal *K* value in the second cluster was found to be 6.

Table 3 shows the results of classification of 101,607 gaits into 7 groups along with the levels of acceleration and angular velocity. In the normal gait group, both acceleration and angular velocity are at average levels. In the abnormal gait 1 group, both values are classified as “Low” level. In the abnormal gait 2 group, acceleration is “Low” level and angular velocity is “High” level whereas the abnormal gait 5 group indicated the opposite. In addition, in the abnormal gait 3 and 4 groups, both acceleration and angular velocity are high (very high or high). Finally, in the abnormal gait 6 group, both values are very low. The number of gaits included in each group is calculated as the probability of the corresponding event. Based on the probabilities of the seven types of gait, a behavioral response distribution was created for each cell.

### 3.3. Comparison of Distribution by Cells with/without an Environmental Barrier

**Figure 5:**
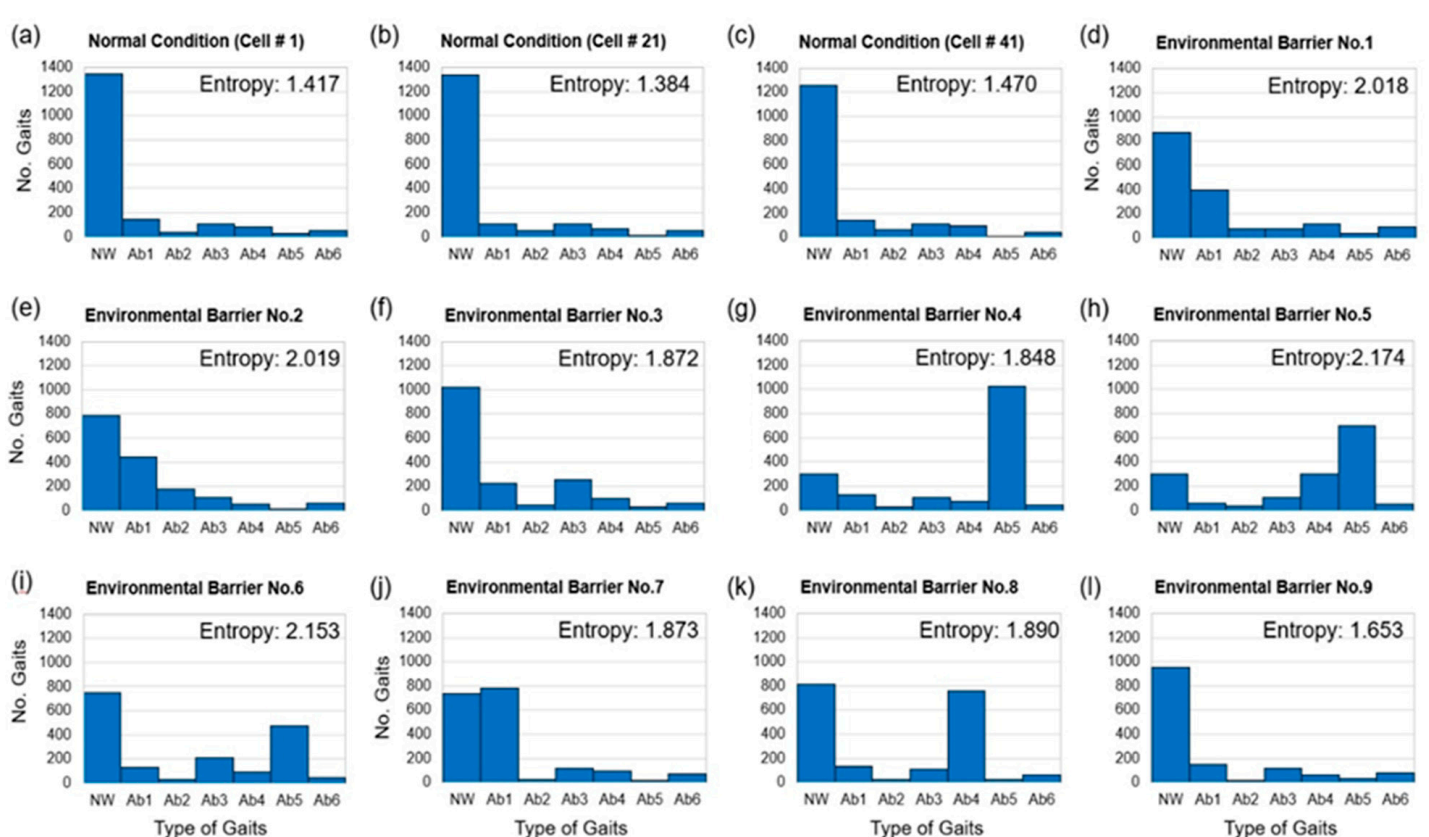
Behavioral response distribution: (**a**–**c**) Behavioral response distribution during normal conditions (Cell Number 1, 21, and 41, respectively) and (**d**–**l**) behavioral response distribution (Cell Number 5, 9, 24, 28, 30, 35, 39, 47, and 60, respectively).

Figure 5 shows the behavioral response distribution for the 12 cells that represent the environmental barrier indicated by the participants and three cells under normal conditions. Figure 5a–c show the distributions corresponding to cell numbers 1, 21, and 41, respectively. In cells that do not contain an environmental barrier, the ratio classified as normal walking by *k*-means clustering is 76.60% (66,141 gaits), and the ratio of behaviors corresponding to AB1–AB6 is 23.40% (20,208 gaits). It was observed that, in cells that do not contain an environmental barrier, the participants walked normally at a very high rate. For cells that contain the environmental barrier as shown in Figure 5d–l, the ratio of normal walking is 42.87% (6541 gaits), and the ratio of behaviors corresponding to AB1–AB6 is 57.13% (8717 gaits).

As Equation (2), it can be seen that the higher the occurrence rate of the rare event, the higher the value of entropy. In order to respond to environmental barriers, pedestrians take the most appropriate or familiar responses to themselves. Due to individual differences, the types of responses that pedestrians can choose can also be diversified, so a high information entropy value is observed at the environmental barrier. On the other hand, in the normal condition, the ratio of normal gait is observed to be very high because the pedestrian has no difficulty in maintaining his/her gait. Therefore, it can be seen that the information entropy is low in normal condition.

On comparing the shape of distribution, it can be observed that the form of behavioral response distribution appearing in each environmental barrier is different. However, behavioral distribution of environmental barrier numbers 1 and 2 is similar. To statistically analyze the shape of each cell, the behavior of cells that do not contain an environmental barrier (NC in Table 4) and nine cells that contain an environmental barrier (EB1–9 in Table 4) are analyzed. Since there are 51 cells that do not contain an environmental barrier, the average information entropy values of seven types of gait corresponding to cells that do not contain an environmental barrier was used to perform a *t*-test for pairwise comparison. Table 4 shows the results of *t*-tests. All *t*-test results except for environmental barrier numbers 1 and 2 (*p*-value = 0.079) show statistical significance (α = 0.05).

### 3.4. Information Entropy Values by Location

**Figure 6:**
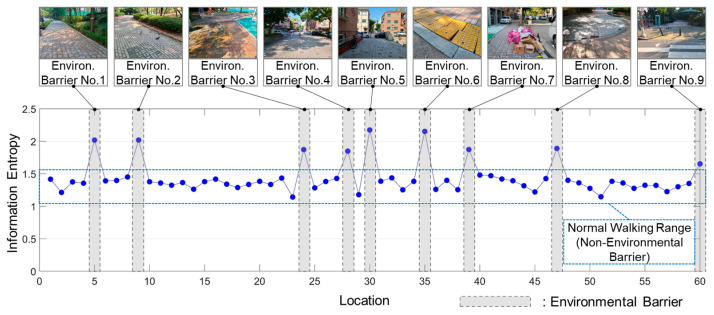
Information entropy value by location.

Information entropy was calculated based on the behavioral response distribution of each cell as presented in the previous section. Figure 6 shows the information entropy values and environmental barriers according to the location. This is consistent with the peak values of the nine environmental barriers and information entropy values suggested by the participants in this experiment. This indicates that environmental barriers result in various reactions from pedestrians.

To confirm the feasibility of the proposed method, the relationship between calculated information entropy values and existence of environmental barriers was measured. The information entropy values of the cell are continuous variables whereas the existence of environmental barriers can be represented as a binary variable (existence as 1 and absence as 0). To investigate the relationship between the existence of an environmental barrier and information entropy in a statistical manner, we have used the point biserial correlation coefficient. This is generally used when one variable is continuous and the other is dichotomous [53,54]. Additionally, the values that relate to the existence of the environmental and information entropy values were calculated using Equation (3) as follows:

(3) $$r_{pb}=\frac{\left(M_{1}-M_{0}\right)\sqrt{n_{1}n_{0}/n^{2})}}{\sqrt{\frac{1}{n}}\sum_{i=1}^{n}\;{\left(X_{1}-\bar{X}\right)}^{2}}$$

where *r_pb_* is the point biserial correlation coefficient, *M*_1_ is the mean value of the continuous variable *X* (information entropy values) for all data points in group 1 (existence of environmental barrier), *M*_0_ is the mean value of the continuous variable *X* (information entropy values) for all data points in group 2 (absence of environmental barrier), *n*_1_ is the number of data points in group 1, *n*_0_ is the number of data points in group 2, and *n* is the total sample size.

Based on the point biserial correlation, the correlation between the existence of an environmental barrier and information entropy values was calculated as 0.759 (α < 0.05, *p* = 0.001) According to previous studies [49,50], a correlation coefficient greater than 0.7 indicates a high degree of correlation. The results showed that the information entropy and existence (presence and absence) of environmental barriers are highly correlated.

Additionally, it was observed that the average value of information entropy of 60 cells was 1.432, maximum value was 2.174, and minimum value was 1.142. Based on the presence or absence of an environmental barrier, the average value of cells that did not contain an environmental barrier was 1.342, maximum value was 1.480, and minimum value was 1.142. However, the average value of cells that consisted of an environmental barrier was 1.945, maximum value was 2.174, and minimum value was 1.653.

Based on the results of the experiments conducted in this study, the threshold for measuring the existence of environmental barriers was set to 1.500. That is, when the information entropy value is less than 1.5, the environment is favorable for normal walking, and when the information entropy value is 1.5 or more, there is a possibility of the existence of an environmental barrier in the corresponding location.

Although the threshold of information entropy that can identify environmental barriers is set to 1.5, this value can vary depending on the size of individual cells and number of gait types constituting the distribution. In this study, each cell had a dimension of 30 m. The reason for choosing 30 m as the cell size was to secure a sufficient number of walks to obtain information entropy in one cell. In general, since one gait has a length of about 1.5 m, it was possible to obtain at least 20 walks in one cell. This is because, if there is a sufficient number of gaits, one or two abnormal gait patterns can be prevented from excessively affecting the information entropy calculation process. Unless the size of the environmental barrier was very large, the range occupied by it at a distance of 30 m was not large. Therefore, even if there is an environmental barrier, many gaits were still classified as normal walking. This is because, the ratio of normal walking varies with the distance of the section, and the value of threshold presented above may change. In addition, seven gait types were used to estimate information entropy. The range of information entropy values is affected by the number of gait types used. Therefore, if the number of gait types is changed, the threshold value also changes.

## 4. Discussion

### 4.1. Contributions of the Suggested Method

The proposed method is used to quantify the behavior of pedestrians in response to environmental barriers using information entropy. Information entropy is calculated using probability distribution. To obtain this distribution, six types of data (3-axis acceleration and 3-axis angular velocity) acquired through the IMU sensor were used to monitor minute changes in the gait process. Six types of data appearing in the gait process were clustered and classified into seven types of gait, which can be also explained as follows: (1) normal gaits are normal walking; (2) abnormal gait 1 is smooth detour; (3) abnormal gait 2 is sharp detour; (4) abnormal gait 3 is jumping over a barrier; (5) abnormal gait 4 is stepping a barrier with caution; (6) abnormal gait 5 is stepping a barrier without caution; and (7) abnormal gait 6 is stop and go. In addition, the reason for using seven types of walking is to include more diverse responses in the information entropy calculation process. Information entropy assumes that rarer events have more information. That is, if only normal gaits occupying the majority and abnormal gaits (just using two clusters) are used, the ability to identify environmental barriers may be reduced. When seven types of gait were used in “3.4 Information entropy values by location” Section, the point biserial correlation coefficient was 0.759 (using seven types of gaits). If six types of abnormal gait are not used and all six abnormal gait types are combined as only one type of abnormal gait, the point biserial correlation coefficient is 0.529 (two types of gait, α < 0.05, *p* = 0.001). Considering these results, using more diverse gait types can be advantageous in identifying environmental barriers. Existing studies have conducted research to identify environmental barriers or hazards using only two normal and abnormal gaits [55,56]. Although barriers or hazards can be identified only with the existing binary classification-based approach, the method proposed in this study shows that more detailed characteristics can be inferred by classifying behaviors into subtypes regarding environmental barriers.

In addition, the two-step *k*-means clustering approach proposed in this study shows that six types of abnormal gait can be distinguished. When clustering only abnormal gaits, the classification performance is higher than when all normal gaits are included. This is because the ratio corresponding to normal gaits is high when calculating the centroid in the process of performing *k*-means clustering for all gaits; hence, an abnormal gait group with a relatively small number of gaits (classified as abnormal gait groups 1–6) of centroids may not be clearly visible. Abnormal gaits can be classified according to their characteristics using two-step *k*-means clustering. As a result, these behavioral characteristics can be reflected in information entropy. Moreover, the gait types classified in this study can be used to construct machine learning or artificial intelligence-based systems in the future. When new data are generated, they can be added to the distribution by measuring the similarity with the already classified data, instead of clustering.

Finally, information entropy based on diverse behaviors is highly correlated with the existence of environmental barriers compared to existing intensity-based approaches and two types of behavior-based approach. There are several reasons why information entropy can be advantageous for identifying environmental barriers. First, individual differences including physical and cognitive characteristics may be an important trigger for causing diverse responses to an environmental barrier. Information entropy can represent this diversity well. Various responses to the external environment mean that the amount of information increases; even if only some of the pedestrians show abnormal responses, they are included in the information entropy calculation process and strongly affect this. Compared with normality or average, a small number of abnormal behaviors can sufficiently influence the information entropy calculation process.

### 4.2. Future Applications and Benefits of the Suggested Method

Although the conventional methods (e.g., surveys and evaluation by experts) for identifying environmental barriers have indicated what environmental elements act as a barrier for pedestrians, there are several disadvantages such as sample bias and time and financial costs. To overcome these disadvantages, wearable device-based approaches have been attempted. Nevertheless, existing studies using wearable devices and this study also face problems, in that a sample of respondents (participants) must be recruited, and participants must attach an IMU sensor. To develop a real-world application, there is a need to replace the IMU sensor with a smartphone. Smartphones include various sensors, and the IMU sensor is also built into the device. In addition, smartphones have the advantage of being possessed by most citizens in their daily life.

In order to change the method proposed in this study to fit the real-world situation, it is necessary to make it possible for pedestrians to provide data on their daily life by carrying a smartphone rather than directly attaching the IMU sensor to their body. If data collection through a smartphone is possible, data of pedestrians can be collected in real time. Real-time data collection will enable continuous monitoring of the walking environment. Despite these advantages, there are several hurdles that must be overcome for a people-centric sensing approach to be feasible, including recruiting and sampling participants [57] and protecting the privacy of participants [27]. Although research related to people-centric sensing is still immature, it is expected that the hurdles will be addressed in the near future [58].

### 4.3. Limitations and Future Research

Despite the strength and contribution of the method proposed in this study, there are several limitations. Walkability and environmental barriers are a relative concept [59]. Even more, walkability is the result of a combination of physical features, urban design qualities, and individual reactions [9,10]. However, the walkability and environmental barriers in this study do not incorporate all these elements. This study tried to identify features that can make pedestrians’ walking patterns dispersed. In consideration of overall walkability, the high normality of gait patterns may not be suitable for developing an attractive street. For example, a pedestrian’s gait pattern can be dispersed if there are many attractive elements in a street and a pedestrian pays attention to the elements. Therefore, the results of this study are limited to identifying environmental barriers that are usually fixed and inconvenient. Future study needs to investigate a more comprehensive meaning of walkability.

In this study, an experiment was performed by attaching an IMU sensor to the ankle of the participants for gait analysis. In future, the results of this study can be extended to people-centric sensing to promote built environment monitoring of a smart city. Therefore, it is necessary to conduct a behavioral analysis of pedestrians with smartphones rather than attaching a sensor to the ankle. In other words, it is necessary to detect and classify various responses to environmental barriers even while walking with a smartphone in a pocket or in hand. In addition, the Bundang area selected as the experimental site in this study was well managed as a planned city. Hence, it is essential to conduct experiments under various conditions for different types of experimental sites.

## 5. Conclusions

Walking is the most basic physical activity to stay healthy. Therefore, it is essential to create an ideal walking environment by identifying the environmental barriers that can impede walking. Efforts to identify existing environmental barriers are labor-intensive, time-consuming, and discontinuous. Although previous studies have attempted to identify environmental barriers using a wearable sensor and people-centric sensing, they have only focused on the magnitude of responses (e.g., high value of accelerations or EDA) at the barrier and not the reactions of the participants.

In this study, we proposed a method to estimate information entropy by quantitatively measuring various responses to environmental barriers using wearable sensors. To verify the proposed method, we performed an experiment on 64 participants. Based on the data collected from the experiment, seven types of gait were classified according to the responses of the participants through two-step *k*-means clustering. The behavioral response distribution for each location was obtained and information entropy was calculated. The results indicated a high correlation with the presence or absence of environmental barriers. Additionally, it was highlighted that the proposed method is feasible for identifying environmental barriers.

## Figures and Tables

**Table 1:** Summary of characteristics of each section.

Section Number	Description	Length (m)	Avg. Width (m)
1	Well-maintained sidewalk blocks Slight uphill road	171	4.8
2	Well-maintained sidewalk blocks installed for over 10 years	118	2.4
3	Well-maintained sidewalk blocks Slight downhill	275	4.5
4	Well-maintained sidewalk blocks in a park installed for over 10 years	219	2.6
5	Crossing on a six-lane road (with traffic lights)	26	4.8
6	Well-maintained sidewalk blocks Crossings on two-lane roads (no traffic lights)	114	6.2
7	Crossings on four-lane roads (no traffic lights)	11	8
8	Mixed residential and commercial spaces Illegal parking and piled materials on sidewalks	324	1.8
9	Crossings on four-lane roads (with traffic lights)	13	8
10	Well-maintained sidewalks and surrounding facilities	274	4.8
11	Well-maintained sidewalks and surrounding facilities	231	4.8
12	Crossing on a six-lane road (with traffic lights)	26	4.8

**Table 2:** Age and gender of participants.

Age	Male	Female	Total
20 s–30 s	16	13	29
40 s–50 s	8	8	16
Over 60 s	10	7	17
Total	34	28	64

**Table 3:** Characteristics of classified normal gaits and six types of abnormal gait in terms of accelerometer and gyroscope data.

Gait Type	Number of Gaits	Mean of Normalized SVM of Acceleration	Mean of Normalized SVM of Angular Velocity
Normal Gait	72,682	Moderate	Moderate
Abnormal Gait 1	7570	Low	Low
Abnormal Gait 2	2719	Low	High
Abnormal Gait 3	6506	High	Very High
Abnormal Gait 4	5419	Very High	High
Abnormal Gait 5	3407	High	Low
Abnormal Gait 6	3304	Very Low	Very Low

**Table 4:** *t*-test results of pairwise comparison among cells with/without an environmental barrier.

*p*-Value	NC	EB1	EB2	EB3	EB4	EB5	EB6	EB7	EB8	EB9
NC	-	-	-	-	-	-	-	-	-	-
EB1	<0.001	-	-	-	-	-	-	-	-	-
EB2	<0.001	0.079	-	-	-	-	-	-	-	-
EB3	<0.001	<0.001	<0.001	-	-	-	-	-	-	-
EB4	<0.001	<0.001	<0.001	<0.001	-	-	-	-	-	-
EB5	<0.001	<0.001	<0.001	<0.001	<0.001	-	-	-	-	-
EB6	<0.001	<0.001	<0.001	<0.001	<0.001	<0.001	-	-	-	-
EB7	<0.001	<0.001	<0.001	<0.001	<0.001	<0.001	<0.001	-	-	-
EB8	<0.001	<0.001	<0.001	<0.001	<0.001	<0.001	<0.001	<0.001	-	-
EB9	<0.001	<0.001	<0.001	<0.001	<0.001	<0.001	<0.001	<0.001	<0.001	-

## Data Availability

Some or all data, or code generated during the study are proprietary or confidential in nature and may only be provided with restrictions (e.g., anonymized data).
